# Functional Characterization of a Wheat NHX Antiporter Gene *TaNHX2* That Encodes a K^+^/H^+^ Exchanger

**DOI:** 10.1371/journal.pone.0078098

**Published:** 2013-11-01

**Authors:** Yuanyuan Xu, Yang Zhou, Sha Hong, Zhihui Xia, Dangqun Cui, Jianchun Guo, Haixia Xu, Xingyu Jiang

**Affiliations:** 1 College of Agronomy/Key laboratory of Physiological Ecology and Genetic Improvement of Food Crops in Henan Province, Henan Agricultural University, Zhengzhou, China; 2 Hainan Key Laboratory for Sustainable Utilization of Tropical Bioresources/College of Agriculture, Hainan University, Haikou, China; 3 Institute of Tropical Bioscience and Biotechnology, Chinese Academy of Tropical Agricultural Sciences, Haikou, China; CNR, Italy

## Abstract

The subcellular localization of a wheat NHX antiporter, TaNHX2, was studied in Arabidopsis protoplasts, and its function was evaluated using *Saccharomyces cerevisiae* as a heterologous expression system. Fluorescence patterns of TaNHX2-GFP fusion protein in Arabidopsis cells indicated that TaNHX2 localized at endomembranes. TaNHX2 has significant sequence homology to NHX sodium exchangers from Arabidopsis, is abundant in roots and leaves and is induced by salt or dehydration treatments. Western blot analysis showed that *TaNHX2* could be expressed in transgenic yeast cells. Expressed TaNHX2 protein suppressed the salt sensitivity of a yeast mutant strain by increasing its K^+^ content when exposed to salt stress. TaNHX2 also increased the tolerance of the strain to potassium stress. However, the expression of TaNHX2 did not affect the sodium concentration in transgenic cells. Western blot analysis for tonoplast proteins indicated that the TaNHX2 protein localized at the tonoplast of transgenic yeast cells. The tonoplast vesicles from transgenic yeast cells displayed enhanced K^+^/H^+^ exchange activity but very little Na^+/^H^+^ exchange compared with controls transformed with the empty vector; Na^+^/H^+^ exchange was not detected with concentrations of less than 37.5 mM Na^+^ in the reaction medium. Our data suggest that TaNHX2 is a endomembrane-bound protein and may primarily function as a K^+^/H^+^ antiporter, which is involved in cellular pH regulation and potassium nutrition under normal conditions. Under saline conditions, the protein mediates resistance to salt stress through the intracellular compartmentalization of potassium to regulate cellular pH and K^+^ homeostasis.

## Introduction

Salts (mainly NaCl) present in soils can enter the cytosol of plants, where they are toxic to important physiological and biochemical processes, and inhibit plant growth and development, thereby significantly reducing yield. On a world scale, no toxic substance restricts plant growth more than salts; therefore, salt stress presents an increasing threat to plant agriculture [1]. Restricting Na^+^ influx into cells may improve plant growth under salinity stress; however, little is known about the mechanisms that inhibit Na^+^ influx. On the other hand, to tolerate high levels of Na^+^ ions, plants cells must be able to transport Na^+^ out of the cytoplasm to the external medium or sequester it to the vacuole, thus maintaining the cytosolic Na^+^ concentration at a non-toxic level. The plasma membrane Na^+^/H^+^ antiporter SOS1 (salt-overly-sensitive), which is energized by the proton gradient generated by the plasma membrane H^+^-ATPases, mediates Na^+^ tolerance of plants through Na^+^ extrusion [2]–[5]. Sodium transport into vacuoles can be accomplished by the operation of tonoplast-bound NHX proteins, which function as Na^+^/H^+^ antiporters, driven by the electrochemical gradient of protons generated by the vacuolar H^+^-ATPase and H^+^-PPase. The compartmentalization of Na^+^ into vacuoles not only averts the deleterious effects of Na^+^ in the cytoplasm, but also allows the plants to use Na^+^ ions as an osmoticum, helping to maintain an the osmotic potential that drives water into the cell. Thus, NHX antiporters have important roles in the plant’s response to salt stress [6].

Vacuolar Na^+^/H^+^ antiport activity was first directly measured in tonoplast vesicles purified from red beet storage tissue [7]. Recent molecular analyses of the antiporters, and the characterization of factors affecting their activity, have allowed the determination of their function and regulation. The DNA sequences encoding NHX proteins have been discovered in more than 60 plant species, including gymnosperms and dicotyledonous and monocotyledonous angiosperms. The expression of most of these *NHX* genes was induced by NaCl treatment [8]. Following the phylogenetic classification by Saier [9], plant NHXs are classified as members of the NHE/NHX subfamily of Na^+^/H^+^ exchangers, a subgroup of the cation antiporter CPA1 family that is present across all taxonomic groups of eukaryotes. On the basis of protein sequence similarity and subcellular localization, the plant NHX family can be further subdivided into two groups, class I and class II [6], [8], [10]. The class-I NHX proteins are localized in the tonoplast, where they function as (Na^+^, K^+^)/H^+^ antiporters. They are involved in Na^+^ or K^+^ accumulation inside vacuoles for the maintenance of cell turgor to drive cell expansion [11]–[13]. The class-II NHX proteins are found in endosomal vesicles of plants, such as Golgi and prevacuoles. These proteins are involved in the regulation of K^+^ homeostasis, which is essential for normal plant growth and development, and play an important role in the response to salt stress by improving K^+^ accumulation [8]. Overexpression of vacuolar Na^+^/H^+^ antiporter NHX genes suppressed the salt sensitive phenotype of a yeast mutant defective for endosomal/vacuolar Na^+^/H^+^ antiporters and conferred salt tolerance to transgenic plants [14], [15]. The first endosomal NHX proteins to be described in plants were the tomato LeNHX2, Arabidopsis AtNHX5 and AtNHX6 proteins. AtNHX5 and AtNHX6 localize in the Golgi and trans-Golgi network, where they may regulate endosomal pH [16]. LeNHX2 is the only class-II NHX protein whose biological functions have been studied in detail. The encoded protein catalyzes K ^+^/H^+^ exchange, but only effects minor Na^+^/H^+^ exchange in proteoliposomes or plant endosomal membranes [17], [18]. These reports implicate a pivotal function of the NHX family in intracellular compartmentalization of Na^+^ or K^+^ and salt tolerance. The vacuolar Na^+^/H^+^ exchange in *Arabidopsis thaliana* is regulated by the SOS pathway and by interaction with the calmodulin-like protein CaM15 [19], [20]. AtSOS2 indirectly regulates ion transport activity in the vacuolar membrane by interacting with the vacuolar H^+^-ATPase [21].

Wheat is one of the most important crops in the world. As a non-halophyte, its growth is severely inhibited by salt stress. When wheat is exposed to salt, the Na^+^ content of cells increases rapidly. In wheat roots, high rates of Na^+^ efflux were inferred because net uptake was very low relative to unidirectional influx [22]. Extrusion of Na^+^ through the plasma membrane by a wheat Na^+^/H^+^ antiporter (TaSOS1) has been experimentally investigated [23]. The overexpression of the vacuolar NHX antiporter AtNHX1 from *Arabidopsis* improved the salt resistance of wheat [24]. A putative wheat Na^+^/H^+^ antiporter, TNHX1, conferred salt tolerance to transgenic *Arabidopsis* plants [25]. In a previous report, the cloning of TaNHX2 from bread wheat was described, and its expression and function were analyzed in wheat and yeast, respectively. It is suggested that TaNHX2 might play an important role in salt tolerance of the plant through compartmentalizing Na^+^ into vacuoles; however, its functions in Na^+^/H^+^ exchange and localization in plant cells have not been demonstrated [26]. Here, we studied the function of the bread wheat NHX antiporter, TaNHX2, in detail. TaNHX2 is closer to NHX members of class I than of class II. In this study we analyzed its sub-cellular localization in plant cells by transient expression of a GFP fusion protein in Arabidopsis protoplasts, and studied its function by heterologous expression in yeast. We discovered that TaNHX2 is the first class I NHX protein that localizes to endosomal (non-vacuolar) membranes in plants. It mainly functions as an intracellular K^+^/H^+^ antiporter and enables the maintenance of higher K^+^ concentrations in yeast cells under the condition of salt stress like tomato LeNHX2, a characterized class II NHX protein [17], [18]. These results suggested that the functions of plant NHX proteins depend more on their location in cells than on their sequence and structural similarities.

## Materials and Methods

### Plant Materials and Growth Conditions

Wheat (*Triticum aestivum* L. cv. Yumai 2) seeds were surface sterilized with 50% ethanol for 3 min and washed twice with distilled water. They were then germinated and grown in a growth chamber at 13–25°C with a relative humidity 65–70%. Ten-day-old seedlings in 1/2 Hoaglands nutrient solution were treated with 200 mM NaCl or 20% PEG (Polyethyleneglycol 6000). After 0, 3, 6, 9, 12, 24 and 48 h, the roots and leaves of the plants were collected, and washed thoroughly several times with doubly distilled water, blotted dry and immediately frozen in liquid nitrogen and kept at −80°C for RNA extraction.

### Isolation of an TaNHX2 cDNA Clone

Total RNAs were isolated from frozen shoots of wheat plants exposed to 200 mM NaCl for 2 d using TRIzol® Reagent (Invitrogen, Carlsbad, CA, USA), according to the manufacturer’s instructions. The cDNA was synthesized using M-MLV reverse transcriptase (Promega, Madison, WI, USA). The full length cDNA of TaNHX2 was amplified with LA DNA Polymerase (TaKaRa, Otsu, Shiga, Japan). Gene specific primers were designed according to the sequence of TaNHX2 (GenBank Accession No. AY040246.) with *EcoR*I and *Sal*I restriction sites (underlined): Forward: 5′- CAGAATTCATGGGGTACCAAGTGGTGGC -3′; Reverse: 5′- AGGTCGACGCGACGTTCATTCCACGAGTAC -3′. RT-PCR amplification was performed using the DNA Thermal Cycler (ABI 9700; USA). PCR conditions were 94°C for 3 min; followed by 35 cycles of 94°C for 30 s, 57°C for 40 s and 72°C for 2 min; with a final extension at 72°C for 10 min. The amplified fragment was ligated into vector pMD18-T (TaKaRa) and sequenced.

### Transient Expression of Green Fluorescent Protein (GFP) Constructs in Arabidopsis Protoplasts

A full-length cDNA of *TaNHX2* without the stop codon at the 3′ end was synthesized by PCR amplification using a forward primer 5′- ATAAAGCTTATGGGGTACCAAGTGGTGGGC -3′ and a reverse primer 5′- GATGTCGACTTCCACGAGTACGTTCGGATC -3′, resulting in a sequence with *Hind* III and *Sal* I sites (underlined in the above primers) and inserted between the constitutive CaMV 35S promoter and *GFP* gene in a pJIT163-GFP expression vector to generate an in-frame fusion of GFP to the C-terminus of TaNHX2. The PCR product obtained was digested with *Hin*dIII and *Sal*I, and then ligated into the *Hin*dIII and *Sal*I sites of the pJIT163-GFP plasmid polylinker to create recombinant plasmid pJIT163-TaNHX2-GFP for expressing the fusion protein. Positive plasmids were confirmed by restriction analysis, and further verified by sequencing. Mesophyll protoplasts were isolated from 3-week-old wild-type Arabidopsis plants (ecotype Columbia) and transformed with pJIT163-GFP and pJIT163-TaNHX2-GFP, respectively, as described previously (http://genetics.mgh.harvard.edu/sheenweb/protocols_reg. html). Visualization of GFP in the transformed protoplasts was performed using a confocal laser-scanning microscope after the protoplasts were incubated at 23°C for 6–18 h.

### Real-time Quantitative PCR

Real-time PCR was performed using platinum Taq DNA polymerase (Invitrogen) and SYBR-Green I (Sigma, USA) as the fluorescent reporter in a Roter Gene 2000 (Corbett company, Australia). Total RNA was isolated from frozen roots and leaves using the TRIzol Reagent (Invitrogen) and first strand cDNA was synthesized using the MMLV reverse transcriptase (Promega). Gene specific primers were designed for a 119 bp fragment of TaNHX2. The primer pair was 5′-CTCCAGAACTTCGATCCTAACC -3′ (forward primer) and 5′-GCACTAAGCAATCCAGTAAACAC -3′ (reverse primer). The primer pair for the housekeeping gene actin (GenBank Accession No. GI:48927617) was 5′-GTTCCAATCTATGAGGGATACACGC-3′ (forward primer) and 5′-GAACCTCCACTGAGAACAACATTACC-3′ (reverse primer) with an amplification length of 422 bp. The PCR conditions were 94°C for 3 min; followed by 35 cycles of 94°C for 10 s, 55°C for 30 s and 72°C for 15 s; followed by 7 min at 72°C. Serial dilutions of cDNA were used to make a standard curve to optimize the amplification efficiency. All reactions were performed in triplicate. Melting curves of the reaction products were generated and fluorescence data were collected at a temperature above the melting temperature of non-specific products. The identity of the amplified fragment was confirmed by gel electrophoresis and sequencing. The values of the expression in each sample relative to the standard curve were calculated. The resulting data were normalized by dividing the value of the expression of TaNHX2 in a sample by the value of expression of actin in that sample. The values of relative expression of the TaNHX2 gene in the leaves and roots of wheat plants without salt (200 mM NaCl) or dehydration (20% PEG) treatments (0 h) were arbitrarily set to 100, and values of the relative expression of the gene in the leaves and roots at different time points (3, 6, 9, 12, 24 and 48 h) of salt or dehydration treatments were expressed as percentage of the values at 0 h.

### Yeast Strains and Plasmids

*Saccharomyces cerevisiae* strains AXT3K (Δ*ena1*::*HIS3*:: Δ*ena4*, Δ*nha1*::*LEU2*, Δ*nhx1*::*KanMX4*), Δvnx1 (Δvnx1::KanMX4), OC02 (Δnhx1Δ::HIS3, Δvnx1::KanMX4) and W303 have been described previously [23], [27]. Yeast expression plasmids pDR195 (PMA1 promoter) and pYES2 (Gal promoter) have been described elsewhere [13], [23].

### Plasmid Construction

A RGSH6 histidine tag was inserted into the yeast multicopy vector pYES2 containing the yeast *GAL1* promoter and transcriptional terminator. For this, oligonucleotides with sequence 5′-CTCGAGGATCGCATCACCATCACCATCACTGAA-3′ (forward) and 5′-ACTAGTTCAGTGATGGTGATGGTGATGCGATCC-3′ (reverse), encoding a RGSH6 tag followed by a stop codon after the last histidine and flanked by *Xho*I and *Spe*I restriction sites (underlined), were ligated between the *GAL1* promoter and the terminator of pYES2, resulting in plasmid pYES2-His. The *TaNHX2* gene was amplified from plasmid pMD18-T containing TaNHX2 ORF, using a forward primer 5′- ATGGATCCATGGGGTACC AAGTGGTG -3′ and a reverse primer 5′- GTGCGGCCGCTTCCACGAGTACGTTCGG -3′, resulting in a sequence with Bam I and Not I sites (underlined) and without a stop codon at the 3′ end. This *TaNHX2* fragment was ligated into plasmid pYES2-His, giving rise to plasmid pYES2-TaNHX2-His. For cation/H^+^ exchange activity tests, the TaNHX2 gene was subcloned into pDR195, resulting in plasmid pDR195-TaNHX2. The two constructs were verified by restriction enzyme digestion and sequencing. The resulting plasmid pYES2-TaNHX2-His and control plasmid pYES2, as well as pDR195-TaNHX2 and control plasmid pDR195, were transformed into yeast strain AXT3K or OC02 by the polyethylene glycol-lithium acetate method, respectively.

### Functional Assays of TaNHX2 using Yeast Mutants

The functional characterization of TaNHX2 was investigated by yeast complementation tests. The full-length cDNA of TaNHX2 with a histidine tag was cloned in the yeast expression vector pYES2, and transformed into the yeast mutant strain AXT3K, which is a derivative of W303. AXT3K is very sensitive to salt stress because it is defective in major Na^+^ transporters in the plasma membrane and endosomal membranes. Yeast strains W303, AXT3K transformed with empty vector pYES2, and AXT3K transformed with pYES2-TaNHX2-His were grown in selective APG medium (10 mM L-arginine, 8 mM phosphoric acid, 2% galactose, 2 mM MgSO_4_, 1 mM KCl, 0.2 mM CaCl_2_, plus trace elements and vitamins) to saturation. These precultures were diluted 50-fold, after which 10 µl of serial (10^−1^) dilutions were spotted on APG plates containing 30 and 60 mM NaCl or 0.25 and 0.5 M KCl, as indicated. The plasmid pDR195-TaNHX2 and control plasmid pDR195 were transformed into yeast strain OC02, respectively. After transgenic OC02 and vnx1 mutant yeast cells grew to saturation in YPD medium (1% yeast extract, 2% peptone, 2% glucose), serial dilutions of the strains were spotted on YPD plates with or without 30 and 50 µg of hygromycin B supplementation. Growth was recorded after incubation for 3–5 days at 30°C.

### Isolation of Microsomal Membranes

AXT3K cells with pYES2-TaNHX2-His tag or empty pYES2 were first cultivated in 10 mL APG medium containing selected amino acids and galactose at 30°C to saturation, and then transferred into 1000 ml APG medium and cultured overnight at 30°C with shaking (200 rpm). The cells were harvested, washed and broken in 150 mM Tris-HCl (pH 8.0), 150 mM KCl, 15 mM EDTA (pH 8.0), 2 mM DTT, 20% glycerol, 1 mM PMSF, 1 mM pepstatin A and protease inhibitor cocktail (Sigma) by vortexing with glass beads. Extracts were centrifuged at 1500×g for 5 min to pellet cell debris, and the supernatants were centrifuged at 100,000×g for 30 min to obtain a microsomal fraction. The pellet was resuspended in 150 mM Tris-HCl (pH 7.5), 15 mM EDTA (pH 8.0), 2 mM DTT, 20% glycerol, 1 mM PMSF, 1 mM pepstatin A and frozen in liquid nitrogen and stored at −80°C until use.

### Determination of Intracellular K^+^ and Na^+^ Contents

Yeast cells were grown in 2500 ml APG medium in the presence or absence of 20 mM NaCl at 30°C with shaking (200 rpm). The cells were harvested by centrifugation (5 min at 3000×g) when the culture reached an OD_660_ nm of 0.25±0.01, then washed twice with 10 ml of ice-cold 10 mM MgCl_2_, 10 mM CaCl_2_, 1 mM HEPES. Washed cells were oven-dried at 85°C for 48 h and then weighed, before being digested with 1 mL HNO_3_ for more than 4 h. Finally, after the samples were suitably diluted with distilled water, potassium and sodium ion contents were determined by an atomic absorption spectrometer (AA-670, Shimadzu Corporation, Kyoto, Japan).

### Isolation of Tonoplast Vesicles from Yeast

Tonoplast vesicles were isolated from transgenic or control yeast strain OC02 as described previously [28], [29], with the following modifications. Yeast cells were first cultivated in 300 mL AP medium containing selected amino acids at 30°C for 1 d with gentle shaking (200 rpm). The cells were then transferred into 2,000 mL YPD medium and cultured at 30°C with shaking (200 rpm) until the culture reached an A_600_ of 2 to 4. Yeast cells were harvested by centrifugation at 1,500×g for 5 min. The pellet was resuspended in 5 mM Tris-HCl (pH 7.5), 700 mM sorbitol to an A_800_ of 6. The volume of the suspension was then measured, mixed with 0.25 volumes of the same medium containing lyticase (300 units/mL) and incubated at 30°C for 90 min with gentle shaking (100 rpm). After lyticase treatment, the resulting protoplasts were recovered by centrifugation at 3,000×g for 5 min, washed twice with 1 M sorbitol, and centrifuged again. All subsequent manipulations were carried out at 0–4°C. The pellet of spheroplasts was then suspended in 30 mL buffer A (10 mM MES-Tris (pH 6.9), 0.1 mM MgCl_2_, 12% Ficoll), homogenized in a loosely fitting dounce homogenizer, and centrifuged in a swinging bucket rotor at 4500×g for 5 min. Subsequently, 5 mL of the supernatant was transferred to centrifuge tubes and 5 mL buffer A was gently layered on top. The tubes were centrifuged in a swinging bucket rotor at 60,000×g for 30 min. The white layer at the top of the tubes was collected and resuspended in buffer A, and 5 mL volume of the suspension was then transferred to centrifuge tubes. This was overlaid with 5 mL buffer B (10 mM MES-Tris (pH 6.9), 0.5 mM MgC1_2_, 8% Ficoll) and then centrifuged at 60,000×g for 30 min. The white layer at the top of the gradient was collected and resuspended in an equal volume of 2×buffer C (10 mM MES-Tris (pH 6.9), 0.5 mM MgC1_2_). An equal volume of buffer C was then added and the mixture centrifuged at 60,000×g for 30 min. The pellet was resuspended in 5 mM Tris-MES (pH 7.5), frozen in liquid nitrogen and stored at −80°C.

### Western Blotting

For western blotting, the microsomal membrane fraction (50 µg of protein) and tonoplast fraction (20 µg of protein) were resolved on 10% SDS-PAGE, respectively, and subjected to immunodetection as previously reported [13]. Immunodetection of histidine tagged TaNHX2 was carried out with a monoclonal antibody raised against the RGSH4 epitope (Sigma).

### Cations/H^+^ Exchange Assay

Tonoplast vesicles were used for the transport assays. The formation of ΔpH was established by the activity of the vacuolar H^+^-ATPase and cations/H^+^ exchange activity was measured as a cation-induced dissipation of ΔpH, with the pH value sensitive fluorescent probe quinacrine in the following reaction mixture (1 mL): 5 µM quinacrine, 50 mM Tris-HCl (pH 7.5), 5 mM MgCl_2_, and 50 µg vacuolar proteins. The reaction mixture was placed in a fluorescence spectrophotometer (Hitachi F-2500, Kyoto, Japan) and equilibrated in the dark with stirring for 1 min before fluorescence measurement. The assay was initiated with the addition of 3 mM ATP. When the ΔpH reached a steady state, equal amounts of different sulfate stock solutions were added to the reaction mixture. To determine initial rates of cation/H^+^ exchange, the change of relative fluorescence was measured during the first 30 s after the addition of these salts. Specific activity was calculated by dividing the initial rate of fluorescence recovery, expressed as a ratio of the preformed pH gradient, by the mass of plasma membrane protein in the reaction and time (ΔF% min^–1^ mg^–1^ protein, where ΔF% = (F30–F0)×100%/(Fmax–Fmin)). The change of pH value was measured at excitation and emission wavelengths of 430 and 500 nm, respectively.

### Protein Content Determination

Protein contents of microsomal membrane and tonoplast preparations were determined by the Bradford procedure [30] using BSA as a standard.

### Statistical Analysis

The results were statistically analyzed using the two-tailed Student’s *t*-test. Data are presented as means ± SE and differences with a *P-*value <0.05 were considered significant.

## Results

### Molecular Description and Subcellular Localization of TaNHX2

**Figure 1:**
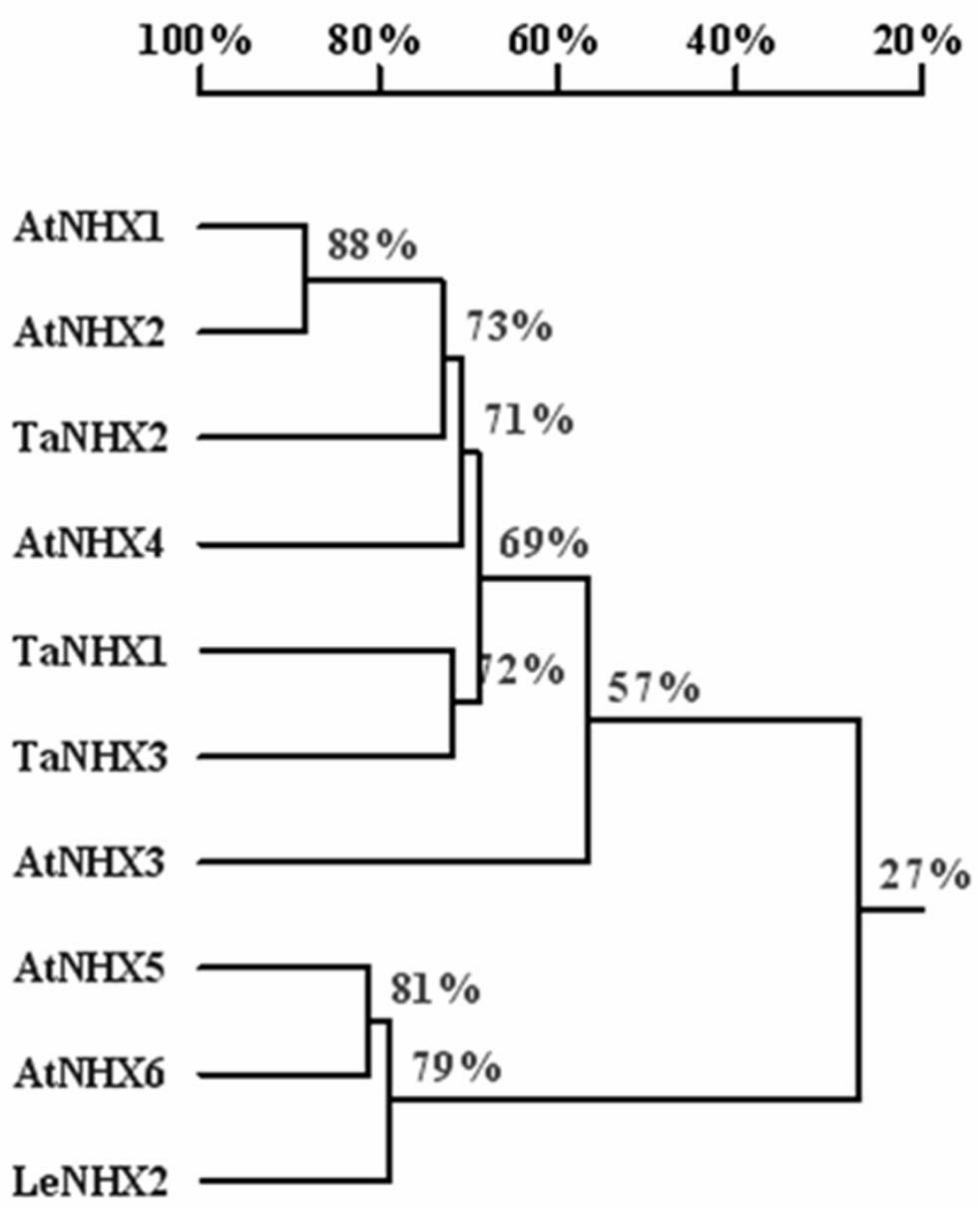
Structural analysis of TaNHX2 protein. Phylogenetic tree of NHX transporter members from wheat, tomato and Arabidopsis. The tree was inferred using the UPGMA method. The alignment is based on the total amino acid sequences. Evolutionary distances were computed by the neighbor joining method. The scale bar indicates the distances as calculated from the multiple alignment. Bootstrap values are indicated at the nodes of the tree and are expressed as percentages.

**Figure 2:**
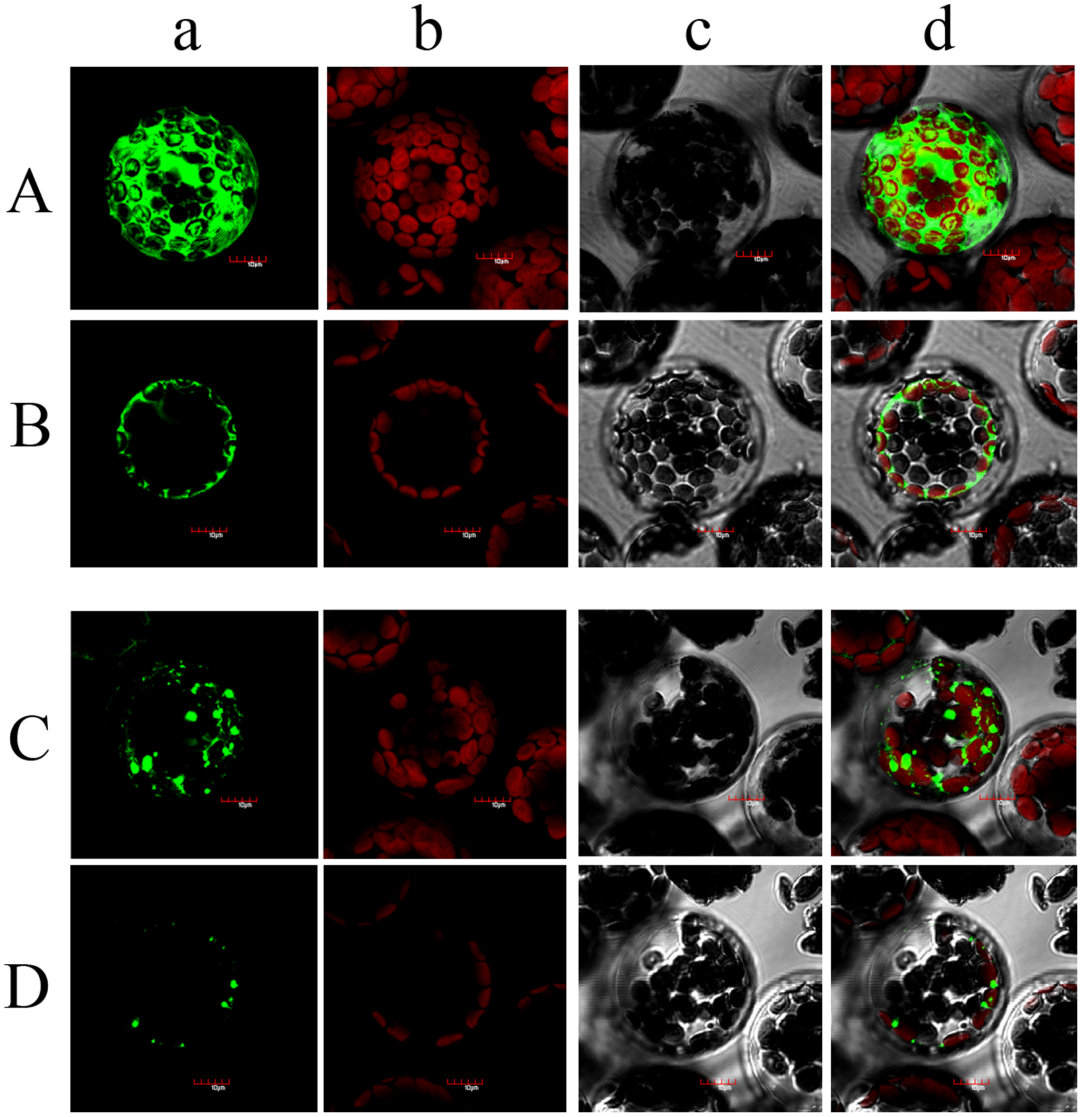
Fluorescence patterns of GFP fusion proteins in plant cells. Arabidopsis protoplasts were transformed with the plasmid pJIT 163 containing GFP alone (A and B), or pJIT 163-TaNHX2-GFP (C and D). The fluorescence images are shown in tridimensional (A and C) and planar (B and D) patterns respectively. Fluorescence microscopic image of GFP (a), fluorescence microscopic image of chloroplasts (b), transmitted light microscopic image of a protoplast (c) and an image of the overlay of the GFP and chloroplast fluorescence and transmitted light (d).

The open reading frame of TaNHX2 gene encodes a protein of 538 amino acids. Eleven putative transmembrane domains in the protein were predicated by TMHMM software (www.cbs.dtu.dk/services/TMHMM/). Multiple alignments of the deduced amino acid sequences of TaNHX2 with two other wheat NHX antiporters, one tomato NHX antiporter and six Arabidopsis NHX antiporters showed that sequence identities between them are 66.24% with TaNHX1 (AAK76737), 69.82% with TaNHX3 (also named TaNHX1 in GenBank, AAS17949), 26.51% with LeNHX2 (AJ306631), 70.17% with AtNHX1 (NM_122597), 71.95% with AtNHX2 (NM_111375), 53.86% with AtNHX3 (NM_124929), 64.33% with AtNHX4 (NM_111512), 27.62% with AtNHX5 (NM_104315) and 27.17% with AtNHX6 (NM_106609) using DNAMAN 6.0 software (Fig. 1). Phylogenetic analysis showed that the three wheat NHX antiporters were closer to Arabidopsis AtNHX1-4 which localize in the tonoplast [11], [12], [31], [32] compared with Arabidopsis AtNHX5-6 and tomato LeNHX2, which are endomembrane-bound proteins [16], [17]. Of them, TaNHX2 was closest to AtNHX1, whose functions have been well studied. We verified the subcellular localization of TaNHX2 using a TaNHX2-GFP fusion protein in Arabidopsis protoplasts. As seen from the planar images (Figure 2B and 2D), central vacuoles were very large, such that the cytoplasm was pushed into a small area between the plasma membrane and tonoplast, where many chloroplasts were located. When GFP was expressed alone, the typical fluorescence of soluble GFP was observed in the cytosol (Figure 2A and 2B). Green fluorescence of TaNHX2-GFP was seen in discrete regions, and appeared to be present in small vesicles but not in the chloroplasts (Figure 2C and 2D). The fluorescence was clearly different from the pattern observed for the tonoplast-localized proteins AtNHX1 and AtNHX2 [12], [17], but was very similar to LeNHX2, AtNHX5 and AtNHX6, which are located in the endomembranes, such as pre-vacuolar or Golgi membranes [16], [17]. These results suggested that TaNHX2 shows higher sequence similarity to vacuolar-bound NHX proteins, but localizes to endomembranes. This would indicate that similar isoforms might have different locations or functions in different plant species.

### Expression of the TaNHX2 in Wheat Plants

**Figure 3:**
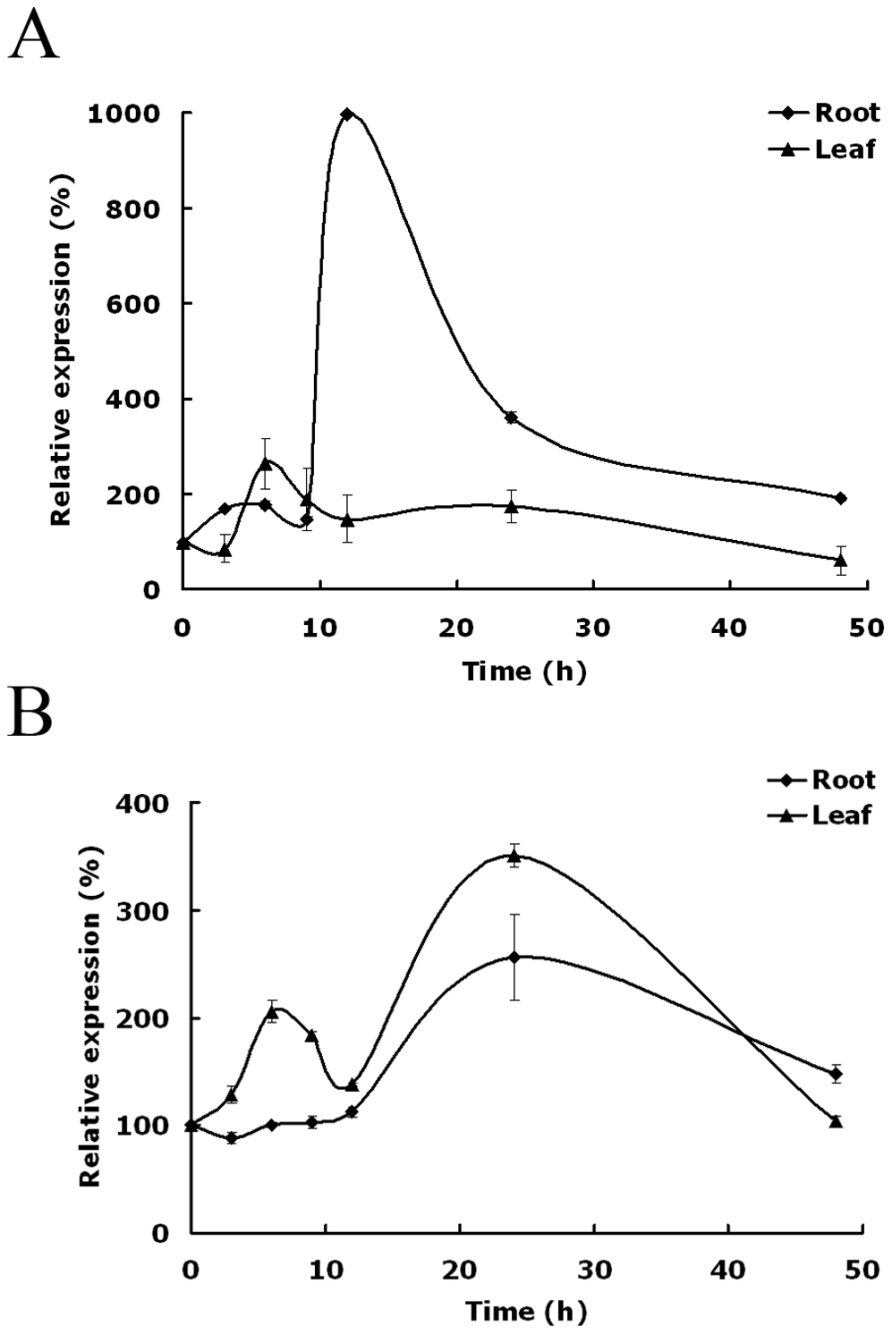
Relative abundance of TaNHX2 mRNA in wheat plants. Ten-day-old plants were transferred to nutrient solution containing 200 mM NaCl or 20% PEG. The expression of TaNHX2 was studied in the roots and leaves at different time points (0, 3, 6, 9, 12, 24 and 48 h) of salt treatment (A) or PEG treatment (B) by real-time PCR. The values of expression at the beginning of salt treatment or PEG treatment (0 h) were arbitrarily set to 100. The expression levels of other time points of salt treatment or PEG treatment were expressed as percentages of the value at time zero. Values are the mean ±SE of three replicates.

The expression of *TaNHX2* was studied in the roots and leaves of wheat plants by real-time PCR. The data presented in Figure 3A shows that the expression level of TaNHX2 in the roots of plants exposed to 200 mM NaCl exhibited the following pattern: the expression level showed an initial small increase, which was maintained until about 9 h after treatment, after which it increased sharply and reached its highest level at 12 h after NaCl treatment. At that point, the expression level was about 10 times higher than that at 0 h. Although the mRNA abundance then decreased, the final expression level at 48 h after stress imposition was about 2 times that at 0 h. The expression level in leaves began to increase after NaCl treatment, reaching its highest expression level at 6 h (2-fold higher than that at 0 h), and then gradually decreased back to basal level at 48 h after exposure to NaCl treatment. To determine whether the expression of TaNHX2 was also induced by dehydration, 10-day-old plants were transferred to hydroponic solution supplemented with 20% PEG (Figure 3B). The expression level in roots remained fairly constant for 9 h after exposure to dehydration stress, and then increased and reached its highest level at 24 h, finally gradually decreasing to an mRNA level at 48 h that was 1.5 times that at 0 h. The expression of TaNHX2 in leaves also was influenced by dehydration. Its expression level increased gradually, and then began to decrease, but the expression level suddenly increased sharply again, reaching its highest level at 24 h, before decreasing back to the basal level at 48 h after dehydration treatment. Thus, it appears that osmotic stress is involved in the upregulation of TaNHX2 in addition to an ion-specific signaling component in wheat.

### Expression of the TaNHX2 Protein in Transgenic Yeast Cells

**Figure 4:**
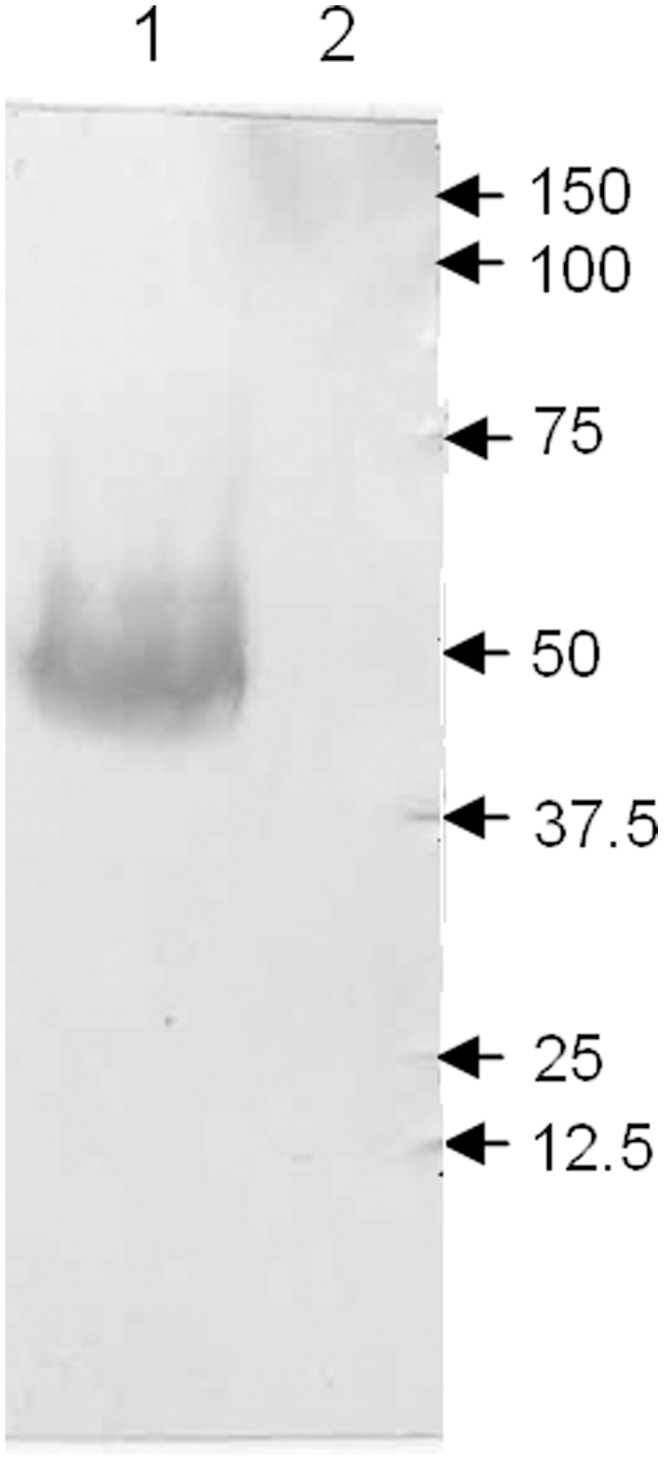
Immunodetection of histidine-tagged TaNHX2 in the microsomal membrane fraction. Microsomal membranes were isolated from yeast cells transformed with empty vector pYES2 and transgenic yeast cells with histidine tagged TaNHX2. 50 µg of microsomal membrane protein was separated electrophoretically and subjected to western blotting with a monoclonal antibody raised against the RGSH4 tag, as described in Materials and Methods. **Lane 1**, the membrane proteins from transgenic yeast cells with pYES2-TaNHX2-His. **Lane 2**, the membrane proteins from yeast cells transformed with empty vector pYES2.

Microsomal membranes were isolated from yeast strain AXT3K expressing or not expressing the histidine tagged TaNHX2 protein. Tagged polypeptide was detected by western blotting using a monoclonal antibody raised against the RGSH4 epitope. A cross-reactive band was observed at a molecular weight of about 50 kDa in transgenic cells. The signal in control microsomes was not detected (Figure 4). This indicated that the immunoreactive band was the TaNHX2-His tagged protein.

### Yeast Functional Complementation

**Figure 5:**
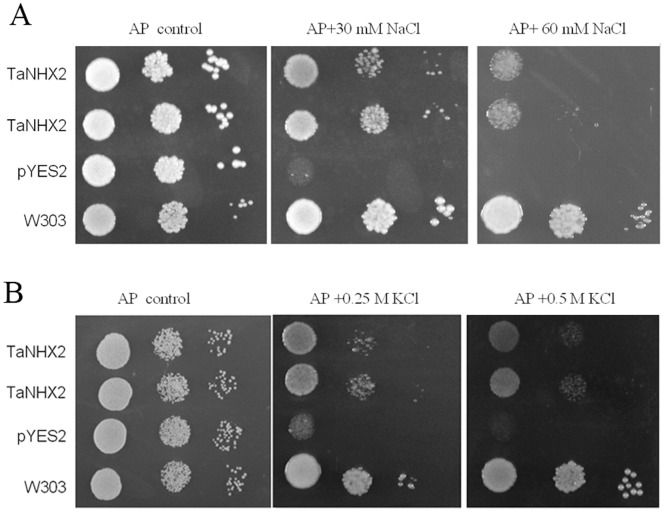
Enhanced salt tolerance of transgenic yeast cells expressing TaNHX2. Yeast cells of recombinant strains, mutant and wild-type were grown to saturation in APG medium. Ten microliters of serial decimal dilutions were spotted onto plates of APG medium supplemented with 0 (control), 30 or 60 mM NaCl (A) and 0.25 or 0.5 M KCl (B) as indicated. Plates were incubated at 30°C for 3-5d. TaNHX2: mutant strain AXT3K transformed with plasmid pYES2-TaNHX2; pYES2: mutant strain AXT3K transformed with the empty plasmid pYES2; W303: wild-type strain W303.

The recombinant plasmid pYES2–TaNHX2-His was obtained by inserting the *TaNHX2-His* fragment into pYES2 between the GAL1 promoter and the CYC1 terminator. The plasmid pYES2–TaNHX2-His was then transferred into the yeast strain AXT3K, in which the major endogenous sodium transporter are disrupted, to test the function of *TaNHX2*. AXT3K transformed with empty vector pYES2 was sensitive to 30 mM and 60 mM NaCl (Figure 5A) as well as 0.25 M and 0.5 M KCl (Figure 5B). The responses of mutant strain AXT3K and AXT3K transformed with empty vector pYES2 to NaCl and KCl stresses were similar (data not shown). *TaNHX2* expression could complement the sensitivity of AXT3K to NaCl and KCl stresses.

**Figure 6:**
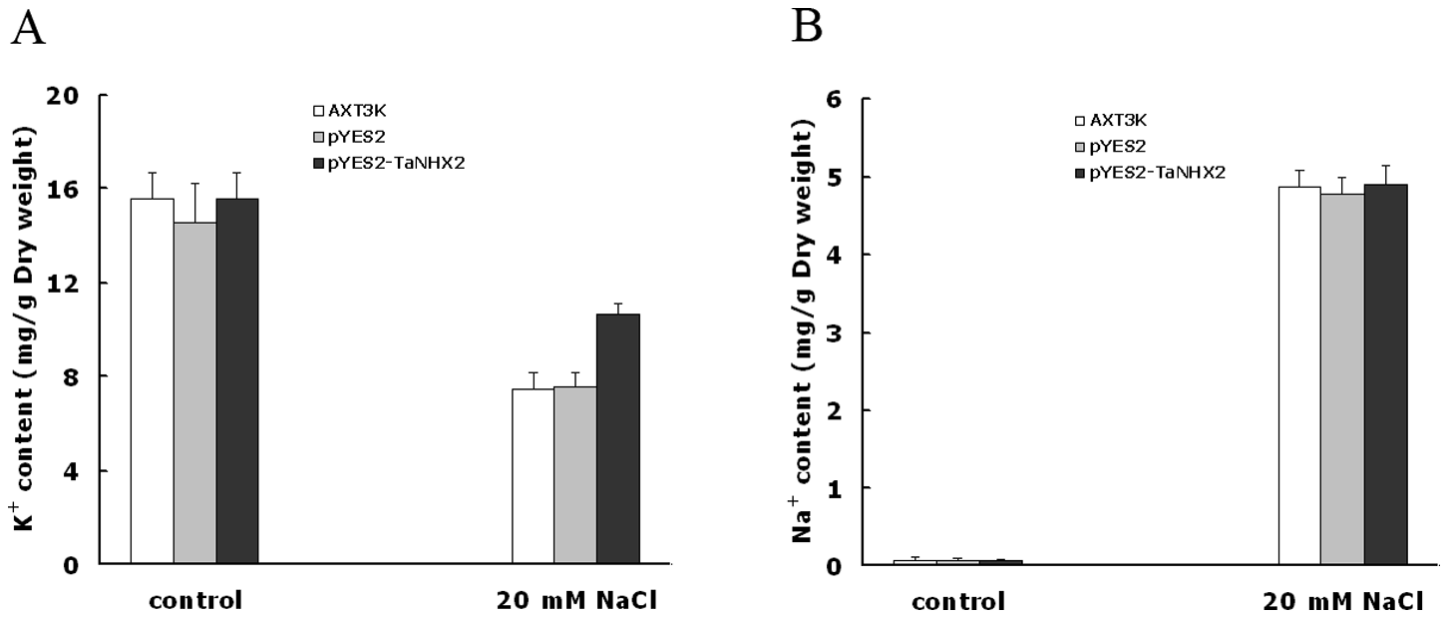
K^+^ and Na^+^ contents in yeast cells. K^+^ (A) and Na^+^ (B) contents were measured in yeast cells grown in APG medium in the absence (control) or presence of 20 mM NaCl by atomic absorption spectrometry, as described in Materials and Methods. The results were analyzed statistically using the two-tailed Student’s *t*-test. Values are the mean ± SE of three replicates and differences with a *P-*value <0.05 were considered significant. AXT3K: untransformed mutant strain AXT3K; pYES2: mutant strain AXT3K transformed with the empty plasmid pYES2; pYES2-TaNHX2: mutant strain AXT3K transformed with plasmid pYES2-TaNHX2.

The Na^+^ and K^+^ contents in untransformed AXT3K, AXT3K transformed with empty vector pYES2 and transgenic cells with TaNHX2 were analyzed using an atomic absorption spectrophotometer (Fig. 6). Without NaCl stress, Na^+^ and K^+^ contents were nearly same in the cells transformed with empty vector and TaNHX2 transgenic cells (*t* test, p = 0.87 and 0.71, respectively). On exposure to 20 mM NaCl treatment, the Na^+^ content increased and K^+^ content decreased in the cells transformed with empty vector and TaNHX2 transgenic cells; intracellular Na^+^ content was similar in both cell types (*t test*, p = 0.26); interestingly, TaNHX2 expression in transgenic cells caused a significantly smaller decrease in K^+^ content compared with cells transformed with the empty vector (*t* test, P<0.01). The contents of Na^+^ and K^+^ in yeast cells untreated and treated with NaCl were not influenced by empty vector pYES2 compared to untransformed AXT3K (*t* test, P>0.05). The results indicate that the TaNHX2 protein might not be involved in changes in sodium concentration, but might contribute to potassium accumulation in transgenic cells.

### Cation/H^+^ Transport Assay

**Figure 7:**
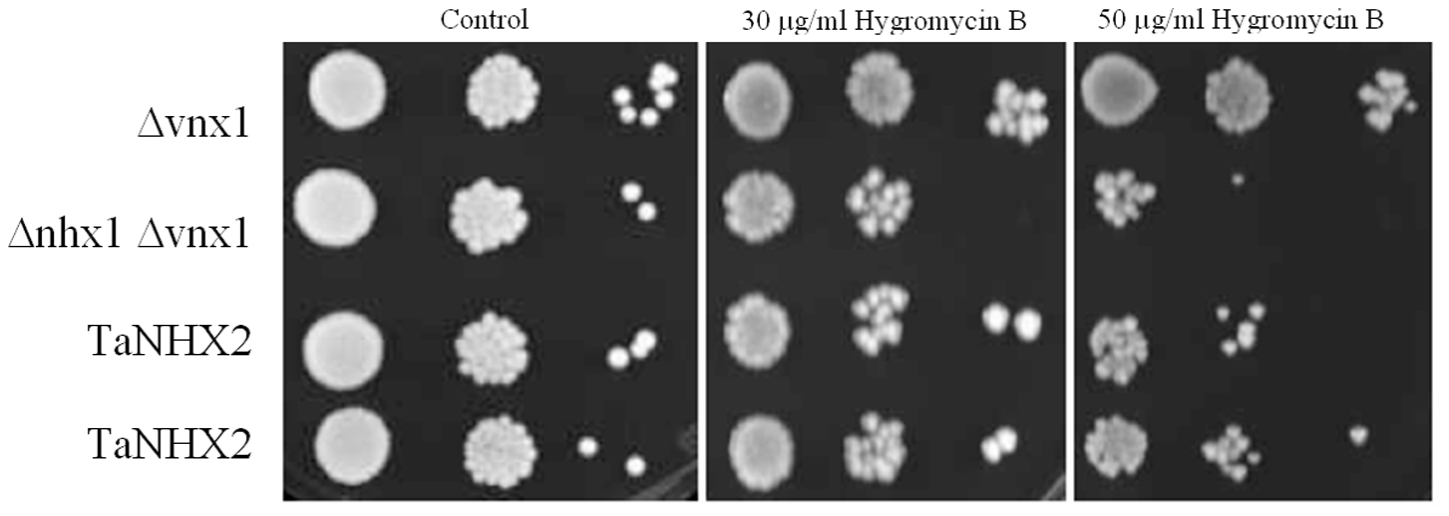
Comparison of yeast cell growth upon hygromycin B treatment. Serial dilutions of the strains were grown on YPD plates containing 0 (control), 30 and 50 µg/ml hygromycin B. Δvnx1: vnx1 mutant yeast strain; TaNHX2: Δnhx1 and Δvnx1 double mutant strain OC02 transformed with plasmid pDR195-TaNHX2; Δnhx1 Δvnx1: Δnhx1 and Δvnx1 double mutant strain OC02 transformed with the empty plasmid pDR195.

**Figure 8:**
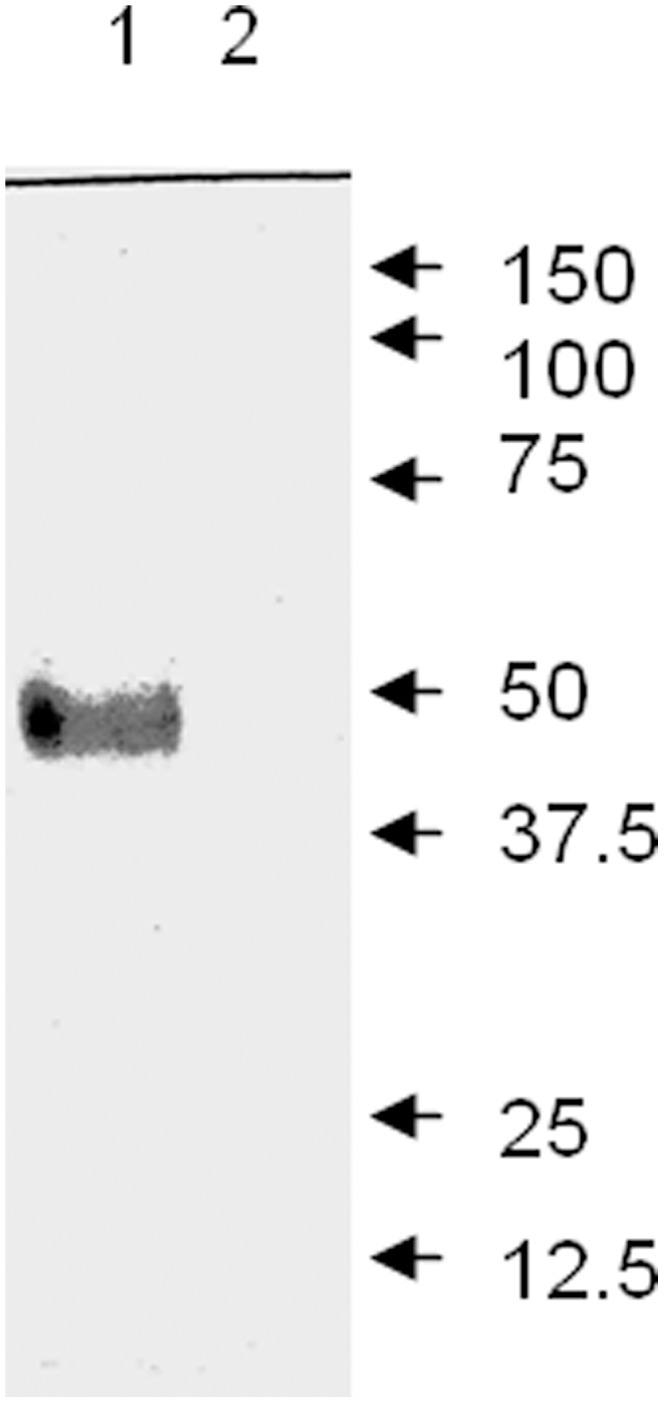
Immunodetection of histidine-tagged TaNHX2 in tonoplasts. The histidine tagged *TaNHX2* cDNA was inserted into yeast expression vector pYes2, and transformed into yeast strain OC02. Tonoplast was isolated from transgenic and untransformed control yeast cells, as described in Materials and Methods. 20 µg of tonoplast protein was separated electrophoretically and subjected to western blotting using a monoclonal antibody raised against the RGSH4 tag, as described in Materials and Methods. **Lane 1**, the tonoplast proteins from transgenic yeast cells with pYES2-TaNHX2-His. **Lane 2**, the tonoplast proteins from untransformed yeast cells.

**Figure 9:**
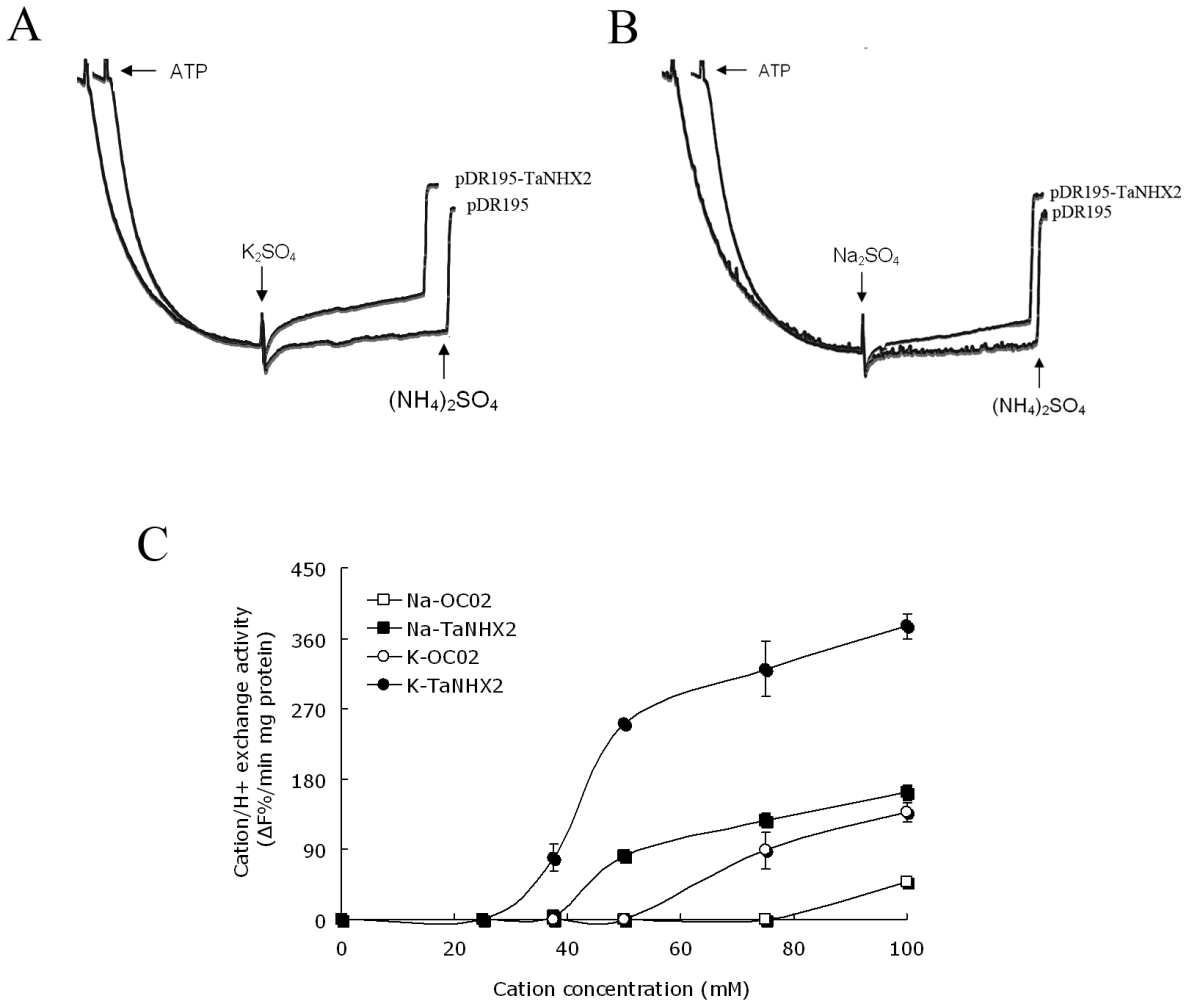
Cation/H^+^ antiport activity in tonoplast vesicles. Fluorescent quenching of quinacrine was used to monitor the acidification of tonoplast vesicles from yeast cells transformed with empty vector pDR195 (pDR195) and transgenic yeast cells expressing TaNHX2 (pDR195-TaNHX2). An inside-acid ΔpH was formed after the addition of ATP to vesicles. Once fluorescence was stabilized, K_2_SO_4_ (A) or Na_2_SO_4_ (B) was added to the cuvette and fluorescence recovery, indicating proton exchange, was monitored for 3 min, after which ΔpH was disrupted by the addition of 25 mM (NH4)_2_SO_4_. Fluorescence is expressed as arbitrary units. C, cation/H^+^ exchange as a function of the concentrations of cations, values are the mean ± SE of triplicate samples. Cation/H^+^ exchange activity is given as the proportion of dissipation of the preformed pH gradient per minute and milligram of membrane protein (ΔF%/mg protein min). Na-OC02: Na^+^/H^+^ exchange activity in tonoplast vesicles from OC02 cells transformed with empty vector pDR195; Na-TaNHX2: Na^+^/H^+^ exchange activity in tonoplast vesicles from OC02 cells transformed with plasmid pDR195-TaNHX2; K-OC02: K^+^/H^+^ exchange activity in tonoplast vesicles from OC02 cells transformed with empty vector pDR195; K-TaNHX2: K^+^/H^+^ exchange activity in tonoplast vesicles from OC02 cells transformed with plasmid pDR195-TaNHX2.

Cation/H^+^ antiporters NHX1 and VNX1 are responsible for the cation/H^+^ exchange in prevacuolar and vacuolar membranes of yeast cells; therefore, an nhx1 and vnx1 double mutant yeast strain lacking monovalent cation/H^+^ exchange activity represents a powerful tool for the study of heterologous expression and functional characterization of endosomal monovalent cation/H^+^ antiporters [27]. Yeast NHX1 is a pre-vacuolar-bound Na^+^/H^+^ antiporter that mediates cation/H^+^ exchange in prevacuoles and possibly in other intracellular compartments; however, its Na^+^ (K^+^)/H^+^ exchange activity could also be detected in tonoplast vesicles isolated from an ScNHX1-transgenic Δnhx1 and Δvnx1 yeast strain OC02, which is almost devoid of backgrounds of K^+^/H^+^ and Na^+^/H^+^ exchanges at the tonoplast [27], suggesting that ScNHX1 is localized at the tonoplast in *ScNHX1* transgenic yeast cells. Growth tests on plates containing hygromycin B showed that TaNHX2 complemented the lack of endogenous ScNHX1 and could increase significantly the tolerance of transgenic yeast cells to hygromycin B stress comparing with control cells (Figure 7). This result is similar to that obtained using its counterparts from other plants, which were localized in pre-vacuolar membranes/tonoplast, like ScNHX1 [18], [33]. This indicates that TaNHX2 may have similar subcellular distribution in transgenic yeast cells as AtNHX1 and ScNHX1, and may be located at the tonoplast in transgenic yeast cells. To test this hypothesis, the histidine tagged *TaNHX2* (*TaNHX2-His*) gene was transformed into the yeast strain OC02, the vacuolar membranes were isolated from untransformed yeast cells and from *TaNHX2-His* transgenic yeast cells and the tonoplast proteins were analyzed by western blotting with an anti-histidine antibody. Figure 8 shows that a band with a molecular weight of about 50 KDa was observed in transgenic yeast cells, which is similar to the protein detected using the total membrane fraction (Fig. 4); however, a signal was not detected in the tonoplast fraction from untransformed yeast cells. These results indicated that TaNHX2 is located at the tonoplast membrane in the transgenic yeast cells. To test whether TaNHX2 functions as a cation/H^+^ exchanger like its counterparts AtNHX1 and ScNHX1 in transgenic yeast cells [27], we isolated tonoplast vesicles from yeast strain OC02 transformed with empty vector pDR195 and TaNHX2-transgenic yeast strain OC02. In the present work, little backgrounds of K^+^/H^+^ and Na^+^/H^+^ exchange activity were observed in the vacuolar membranes of yeast strain OC02 with double mutations of nhx1 and vnx1 (Fig. 9A and B). The tonoplast vesicles from transgenic yeast expressing TaNHX2 exhibited a low Na^+^/H^+^ and higher K^+^/H^+^ exchange activities (Fig. 9A, B and C). Even in absolute terms, that is, subtraction of the backgrounds of Na^+^(K^+^)/H^+^ exchange in vacuolar membrane of yeast cells transformed with empty vector pDR195, K^+^/H^+^ exchange activity was about two times more than Na^+^/H^+^ exchange activity in vesicles derived from yeast cells expressing TaNHX2. These results suggest that TaNHX2 functions mainly as a K^+^/H^+^ antiporter.

## Discussion

The *NHX* genes have been discovered in more than 60 plant species, and the expressions of most of them are induced by NaCl treatment [8]. The expression levels of *TaNHX2* in wheat plants clearly increased in response to salt and dehydration. Thus, it appears that osmotic stress is involved in the upregulation of TaNHX2 in addition to an ion-specific signaling component in wheat. Plant NHX antiporters are members of the sodium proton exchanger subfamily of cation/proton exchangers that are present across all taxonomic groups of eukaryotes, and constitute a subgroup of the cation/proton antiporter 1 (CPA1) family [9], [34]. On the basis of protein sequence similarity, all plant NHXs characterized to date can be classified into two groups: class I and class II [6], [8]. A family of six NHX proteins was identified in Arabidopsis (AtNHX1 to AtNHX6). AtNHX1-4 constitute class I and are 56–87% similar to each other, whereas AtNHX5 and AtNHX6 belong to class II and are 79% similar; however, the similarity among class I and class II NHX proteins is only 21–23%. The phylogenetic tree of 10 characterized NHX sequences shows that TaNHX2 is most closely related to AtNHX1 and AtNHX2 (71% and 72%), but shows only about 27% similar to AtNHX5 and AtNHX6; therefore, it should be a member of the class-I subgroup. All the class-I NHX isoforms studied to date have a vacuolar membrane localization, which appears to be a unique feature of this class. By contrast, all class-II members studied to date are localized in various endosomal compartments of plants. However, our results show that TaNHX2 is not a tonoplast-bound protein, but is located in the endosomal membranes. AtNHX4 has also been found at prevacuolar membranes, but the protein mainly localizes at the tonoplast [32]. To the best of our knowledge, TaNHX2 is the first member of the class I NHX proteins that is reported to localize in endosomal (non-vacuolar) membranes in plants. This would indicate that similar isoforms might have different locations or functions in different plant species.

The budding yeast *S. cerevisiae* is an excellent model for studying the transport properties and physiological functions of ion transporters. The existence of mutant strains lacking their own transport systems provides an efficient tool for the molecular study of alkali-metal-cation transporters from higher eukaryotes when expressed in yeast cells [35]. The nhx1 mutant is sensitive to salt stress, and expression of some plant NHX genes in this yeast mutant can complement the sensitivity [13], [14]. The yeast strain AXT3K, in which major endogenous sodium transporters essential for salt tolerance are disrupted, is very sensitive to salt stress such that it cannot grow on AP plates containing 30 or 60 mM NaCl. Western blotting showed that *TaNHX2* could be expressed in this strain from vector pYES2, and the expressed protein could partly compensate for the sensitivity of AXT3K to salt stress such that cells grew well in 30 mM NaCl, albeit they did not reach the salt tolerance of wild-type yeast cells. Interestingly, TaNHX2 could also increase the tolerance of transgenic yeast to potassium stress. This is the first report that an NHX protein functions as a K-tolerance target. These data indicate that the TaNHX2 antiporter might not only catalyze Na^+^ transport, but also K^+^ transport. However, the sodium content did not change in TaNHX2-transgenic cells as compared with control cells under both normal growth and mild salt stress; on the contrary, TaNHX2 expression caused significantly smaller decrease in potassium content in transgenic cells when exposed to 20 mM NaCl stress. These results suggest that the enhanced salt tolerance of transgenic cells did not involve sodium transport, but potassium accumulation is more important for the tolerance of the cells to salt stress.

Hygromycin B sensitivity is a common phenotype of the yeast nhx1 mutant [36]. TaNHX2 expression in the yeast nhx1 mutant complemented the lack of endogenous ScNHX1 and increased resistance to the polycationic drug hygromycin B, suggesting that TaNHX2 could substitute for ScNHX1 in prevacuolar/tonoplast functions. Yeast NHX1 is Na^+^/H^+^ antiporter that mediates cation/H^+^ exchange in prevacuoles and possibly in other intracellular compartments. However, NHX1’s Na(K)^ +^/H^+^ exchange activity could also be detected in tonoplast vesicles isolated from a ScNHX1-transgenic Δnhx1 and Δvnx1 yeast strain OC02, which is almost devoid of background of K^+^/H^+^ and Na^+^/H^+^ exchange at the tonoplast [27], suggesting that ScNHX1 is located at the tonoplast in transgenic yeast cells. In the present work, TaNHX2 was also found at the tonoplast in the transgenic yeast cells (Fig. 8). To test whether TaNHX2 functions as a cation/H^+^ exchanger, we isolated tonoplast vesicles from OC02 transformed with empty vector pDR195 and TaNHX2-transgenic yeast strain OC02. Transport assays with tonoplast vesicles demonstrated that TaNHX2 could transport K^+^ and Na^+^ into the vesicles, but had a greater capacity for K^+^/H^+^ exchange compared to Na^+^/H^+^ exchange; Na^+^/H^+^ exchange by TaNHX2 could not be induced with concentrations less than 37.5 mM Na^+^ in the reaction medium (Fig. 9). Cytosolic Na^+^ concentrations of 10–30 mM have been estimated in the salt-treated cells by X-ray microanalysis and ion-sensitive electrodes, whereas cytosolic K^+^ often remains above the 60 mM level; thus, the cytosolic K^+^ concentration most likely exceeds that of Na^+^, even under salinity stress [37], [38], [39]. This is in agreement with the ion contents in yeast cells exposed to mild salt stress: the K^+^ content significantly increased, but the Na^+^ content did not change in transgenic yeast cells treated with 20 mM NaCl as compared with control cells. Therefore, the increased accumulation of K^+^ is likely to be a consequence of the activity of TaNHX2. We can speculated that the TaNHX2 antiporter facilitates K^+^ transport into endosomal compartments in exchange for H^+^ into the cytosol in plant cells. The K^+^ ions compartmentalized into the endosomes can act as osmoticum, helping to maintain an osmotic potential that drives water into the cell. The expression level of TaNHX2 was increased by dehydration stress, indicating that the induced K^+^ transporter can help wheat plants obtain more K^+^, and further alleviate the osmotic stress imposed on the plants when exposed to salinity or dehydration. The compartmentalization may prevent Na^+^ toxicity by facilitating cellular K^+^ uptake. The findings presented in this paper will increase our understanding of the relationship between NHX proteins and potassium nutrition in plants.

The function of the plant class-I NHX anitporters localized in the tonoplast has been well studied. The first plant Na^+^/H^+^ antiporter was cloned in Arabidopsis [40]. Expression of AtNHX1 in an nhx1 yeast mutant suppressed its NaCl sensitivity and enhanced the intracellular compartmentalization of Na^+^ [40], [41]. Experiments with vacuolar vesicles isolated from yeast expressing AtNHX1 showed electroneutral Na^+^/H^+^ exchange [42]. Results using leaf vacuoles from transgenic Arabidopsis overexpressing AtNHX1 indicated that Na^+^/H^+^ exchange activity was much higher than in control plants, but did not affect K^+^/H^+^ exchange [43]. These observations suggest that NHX-mediated salt tolerance is a consequence of the accumulation of toxic Na^+^ inside the vacuoles. The compartmentalization of Na^+^ into vacuoles, mediated by tonoplast-bound NHX, not only averts the deleterious effects of Na^+^ in the cytoplasm, but also allows the plants to use Na^+^ ions as an osmoticum helping to maintain an osmotic potential that drives water into the cell. In contrast to many reports on class-I NHX proteins, functional analyses of class-II NHX isoforms is scarce. Subcellular localization studies have only been reported for the tomato LeNHX2, and the Arabidopsis AtNHX5 and AtNHX6 proteins. A translational AtNHX5-GFP fusion protein localized to the prevacuolar membranes of onion cells [8], [16], and the nhx1 yeast mutant expressing AtNHX5 accumulated comparatively less Na^+^ and more K^+^ than class-I antiporters AtNHX1 and AtNHX2 [14]. AtNHX6 was found at endosomal membranes, where it might regulate endosomal pH [16]. In yeast, the histidine-tagged LeNHX2 protein co-fractionated with Golgi and prevacuolar membrane markers. Purified LeNHX2 protein mainly catalyzes K^+^/H^+^ exchange in reconstituted proteoliposomes. Transitory expression in onion epidermal cells of fluorescent fusion proteins showed that LeNHX2 was located in endosomal membranes [17], [18]. The LeNHX2 antiporter overexpressed in Arabidopsis plants functions as an endosomal K^+^/H^+^ antiporter, and increases salt tolerance by improving potassium compartmentalization and regulating pH homeostasis [17]. Presumably, the localization of class-II NHX proteins to endosomal compartments other than the vacuole imposes restrictions on ion selectivity, because proper functioning of the endomembrane system must rely on K^+^/H^+^ exchange for pH regulation to prevent undue accumulation of potentially toxic Na^+^ into the endosomal lumen. Taken together, these data suggest that diverse subcellular localizations of class-I and class-II NHX proteins in plants correlate with distinct substrate-ion specificities and physiological roles. The TaNHX2 antiporter shows higher sequence homology to the class-I proteins than class-II proteins, but its cellular location and biological functions are similar to class-II proteins. AtNHX4 is closer to the class-I NHXs, but the prevacuole-localized protein also functions as a K^+^ transporter [32]. These observations suggest that the functions of plant NHX proteins depend more on their location in cells than their sequence similarities.

Taken together, the results indicate that the TaNHX2 protein is located in the endomembranes of plants. TaNHX2 complemented the salt- and hygromycin sensitive phenotypes of the nhx1 yeast mutant and affected the accumulation of K^+^, but not Na^+^, in intracellular compartments of transgenic yeast cells. TaNHX2 in the tonoplast vesicles of transgenic yeast cells catalyzed K^+^/H^+^ exchange, but very little minor Na^+^/H^+^ exchange in vitro. These data suggest that the endosomal-bound TaNHX2 mainly functions as a K^+^/H^+^ antiporter in plant cells and plays an important role in the response to salt stress by regulating K^+^ homeostasis.
